# Physical, Chemical and Histological Characterization of *Pectoralis major* Muscle of Broilers Affected by Wooden Breast Myopathy

**DOI:** 10.3390/ani11030596

**Published:** 2021-02-24

**Authors:** Rodrigo Fortunato de Oliveira, Juliana Lolli Malagoli de Mello, Fábio Borba Ferrari, Erika Nayara Freire Cavalcanti, Rodrigo Alves de Souza, Mateus Roberto Pereira, Aline Giampietro-Ganeco, Erick Alonso Villegas-Cayllahua, Heloisa de Almeida Fidelis, Maísa Santos Fávero, Lizandra Amoroso, Pedro Alves de Souza, Hirasilva Borba

**Affiliations:** 1 Department of Technology, Universidade Estadual Paulista—UNESP, N/n, Professor Donato Castellane Access Road, Rural Zone, Jaboticabal, Sao Paulo 14884-900, Brazil; julianalolli@zootecnista.com.br (J.L.M.d.M.); fbf_zoo@hotmail.com (F.B.F.); erikanayarac@gmail.com (E.N.F.C.); mateusscj2012@hotmail.com (M.R.P.); eav.cayllahua@unesp.br (E.A.V.-C.); heloisa.a.fidelis@gmail.com (H.d.A.F.); maisa.sfavero@gmail.com (M.S.F.); lizandra.amoroso@gmail.com (L.A.); p.souza@unesp.br (P.A.d.S.); 2 Faculty of Animal Science and Food Engineering, University of São Paulo—USP, 225, Duque de Caxias Norte Avenue, Pirassununga, Sao Paulo 13635-900, Brazil; rodrigo.zootecnista@gmail.com (R.A.d.S.); giampietroganeco@gmail.com (A.G.-G.)

**Keywords:** chicken breast meat, meat quality, myodegeneration, tenderness, wooden breast

## Abstract

**Simple Summary:**

Wooden breast myopathy is a muscle abnormality resulting from the genetic improvement practiced by the poultry industry and which is widespread throughout the planet. Recent research has tried to unravel this myopathy for years and how the quality of the meat is affected, in search of its etiology and the solution to this problem. Unfortunately, the problems related to the myopathies of the affected birds will only be solved when the cause of the abnormality is in fact unveiled and controlled; in the meantime, it is up to us researchers to contribute scientifically to research on this type of meat that is fit for consumption but that, for often presenting non-standard appearance for sale in the form of whole fillets, is discarded.

**Abstract:**

This study aimed to characterize the effects of wooden breast myopathy (WBM) on quality of broiler chicken breast meat. Normal samples (absence of myopathy), moderate-degree samples (hardness only in one area of the breast fillet) and severe-degree samples (hardness throughout the breast fillet) were classified. In macroscopic analysis, the pectoral muscle affected by the WBM showed, in general, pale color with stiff, irregular and reddish regions (suffusions and petechiae), with the presence of white striations. In microscopic analysis, the myopathy was characterized by loss of the polygonal aspect of the muscle fibers. Samples with moderate degree of the myopathy showed greater (*p* = 0.0266) water retention capacity. There was an increase (*p* = 0.004) in total collagen concentration in samples from the severe-degree group 0.29% in normal samples to 0.43% and 0.48% in samples from moderate- and severe-degree groups, respectively. Samples of chicken breast affected by the severe-degree WBM showed lower (*p* < 0.0001) myofibrillar fragmentation index (64.51) and lower (*p* = 0.0002) fat concentration (2.17%) than normal chicken samples (80.45 and 3.79%, respectively). Samples affected by WBM are larger and heavier and present poorer physical quality when compared to normal chicken meat. Histologically it is possible to observe loss of the polygonal aspect of muscle fibers.

## 1. Introduction

The occurrence of wooden breast myopathy (WBM) in broilers has been reported in recent years and is associated with rapid growth and development of birds, but its etiology remains unknown [1,2,3]. The detection of severe cases of WBM can be performed and determined by palpating the *Pectoralis major* muscle. The identification and characterization of WBM in the slaughterhouse are based on the appearance and hardness of the chicken breast [2] and depend on the sensitivity, training and knowledge of the evaluator.

Macroscopic changes are restricted to the *Pectoralis major* muscle, characterized by expansive, pale, hardened areas [2] with or without white striation. Still according to this author, muscles with WBM demonstrate varying degrees of necrotic fibers, fibrosis, small fibers in regeneration, infiltration of immune cells and extensive deposition of fibrillar collagen. This myopathy may alter important parameters for industrial processes.

WBM may present substantial implications for meat quality, but few studies have been found on the characterization of the myopathy. It is not known for sure if the quality and quantity of the meat protein (proportion) and content are affected by such alteration [4]. In the aspect of intact breast meat, many researchers have demonstrated that raw wooden breast fillets exhibited poor WHC and bad hardness [5]. Meanwhile, the physical and chemical qualities, such as the histological composition of chicken meat affected by WBM, mainly of fillets affected to a severe degree, for consumption in natura or in the manufacture of processed products remain uncertain. Given the above, this study aimed to characterize physically, chemically and histologically the breast meat of broilers affected by WBM.

## 2. Materials and Methods

This study was conducted in the Laboratory of Analysis of Animal Origin Foods of the Faculty of Agrarian and Veterinary Sciences of UNESP, Campus Jaboticabal, São Paulo, Brazil (21°08′ S, 48°11′ W, 583 m altitude).

### 2.1. Sample Collection and Experimental Procedure

Sixty samples of breast meat from Cobb MX male broilers bred in traditional intensive system and slaughtered at 45 days old were used. The samples were acquired in a commercial slaughterhouse (State of São Paulo, Brazil) inspected by the Federal Inspection Service. The birds were slaughtered according to the slaughterhouse routine with mechanical deboning of the breast.

The samples, without bone and without skin, were classified by palpation according to the severity degree of the myopathy (moderate (*n* = 20)—hardness only in the cranial region or in the caudal region of the breast fillet; severe (*n* = 20)—hardness over the entire length of the breast fillet). Twenty breast fillet samples classified as normal (absence of myopathy) were also collected and used as a control group for comparison with the affected samples. In order to characterize the affected depth and the evolution of the condition, the collection of muscle fragments of two square centimeters from the affected site and the area beneath it was done in all samples, immediately after the collection and classification of chicken breasts.

After being classified and collected, the samples were transported to the university laboratory under refrigeration conditions (±4 °C) for further quality analysis. After the establishment of rigor mortis (4 h after slaughter), the collected samples were weighed individually and the physical analyses described below were performed. For chemical analysis, sub-samples from each studied group were frozen (−20 °C) for a period of 30 days for further analysis. Physical and chemical analyzes were performed on all chicken breast samples collected (*n* = 20, for each group).

### 2.2. Physical Analysis

The biometrics of the structures were performed with the aid of a ruler for length (cm) and width (cm), and a 6” digital caliper (Zaas Precision, AMATOOLS Commercial e Importadora Ltd.a, Piracicaba, São Paulo, Brazil) for thickness (cm), according to the methodology described by [6].

The color (luminosity—L*; intensity of red—a* and intensity of yellow—b*) was determined immediately after deboning using a Minolta CR-400 colorimeter (Konica Minolta Sensing, Inc., Osaka, Japan) (configurations: diffuse lighting/0 viewing angle, D65 illuminant, specular component included) calibrated to a white standard. The equipment was positioned in three different sites on the external surface of the *Pectoralis major* muscle (which was previously in contact with the skin) and also on three different sites on the internal muscle surface (which was in contact with the sternum bone).

The meat’s pH was evaluated in triplicate with a digital pH meter (Testo 205, Testo Inc., Sparta, NJ, USA) equipped with a penetration electrode, which was inserted into the cranial part of each sample. The water holding capacity (WHC) was determined in triplicate as described in [7].

The samples were cooked in order to evaluate the cooking weight loss (CWL), according to the method described in [8], and the results were obtained by the difference between the initial and final weights, expressed as percentage. The shear force (softness) was performed using the Meullenet–Owens Razor Shear (MORS) method coupled to the Texture Analyzer TA-XT2i in the cooked samples. The shear force was also evaluated using the Warner–Bratzler device coupled to the Texture Analyzer TA-XT2i. From each cooked sample, three sub-samples with a section area of 1 cm^2^ were obtained by being placed with the fibers perpendicularly oriented to the Warner–Bratzler device and subjected to cutting [9]. The force required to shear the samples was expressed in newton.

### 2.3. Chemical Analyses

The chemical composition was determined after physical analysis. For chemical analysis, sub-samples from each studied group were frozen (−20 °C) for a period of 30 days for further analysis. After freezing, the samples were lyophilized (SuperModulyo220, Thermo Fisher Scientific Inc., Waltham, MA, USA) and ground for later determination of protein and mineral matter concentrations, as recommended by [10], 977.14 and 920.153 methods, respectively. The percentage of moisture was determined by the difference between the sample weights before and after lyophilization [8; 950.46 method]. Fat was determined according to the method proposed in [11].

Lipid oxidation was determined in fresh samples right after their arrival from the slaughterhouse by the thiobarbituric acid reactive substances (TBARs) test, according to the methodology described by [12]. Cholesterol was determined after adapting the method described by [13]. After weighing 0.5 g of lyophilized sample in a 50 mL falcon tube, 6 mL of ethanol and 4 mL of 50% KOH were added. The tubes were kept in a water bath with agitation (40 °C) until the samples were completely dissolved and, afterwards, for another 10 min (60 °C). Ten mL of N-hexane was added three times for phase separation and the upper phase of the sample was removed and put into another tube. Afterwards, 3 mL aliquots were pipetted from the upper phase into test tubes and nitrogen gas drying was performed. After drying, 0.5 mL of isopropyl alcohol was added to each tube. The tubes were vortexed, and 3 mL of enzyme reagent was added for blood cholesterol analysis (adapted [13]). Then, the material was kept in water bath (37 °C) for 10 min. Samples were read on a spectrophotometer (Shimadzu UV-1800, Shimadzu Corporation, Kyoto, Japan) with λ equal to 500 nm (according to the kit manufacturer).

Fatty acids were isolated according to the method proposed by [11], which removes the lipid phase from the sample. The esterification of fatty acids was performed by the methylation method [14] and analyzed by a gas chromatograph (Shimadzu 14 B, Shimadzu Corporation, Kyoto, Japan) equipped with flame ionization detector and fused silica capillary column (Omegawax 250, Sigma-Aldrich Japan Co. Ltd., Tokyo, Japan); H2 was used as the carrier gas. The peaks were identified by comparison with the pattern retention times of known composition.

The concentrations of total, soluble and insoluble collagen were quantified in the samples by determining the hydroxyproline amino acid according to procedures recommended by [15,16,17]. The myofibrillary fragmentation index (MFI) was determined as described by [18], being the biuret method [19] used to determine the concentration of proteins in the suspension of myofibrils. The calculation was performed according to the following formula: MFI = optical density × 200.

### 2.4. Histological and Morphological Analysis

For the histological analysis of muscle fiber, two-centimeter cross-sectional samples were taken from the cranio-lateral region of *Pectoralis major* muscle of ten birds and then stored for 24 h in plastic containers with Bouin’s fixing solution. Subsequently, the samples were washed in 70% alcohol (to remove the fixative) and dehydrated in a series of increasing ethanol concentration (70%, 80%, 90% and 100%). Then, the material was diaphanized in xylol and infiltrated in histological paraffin. Semi-serial histological sections of five micrometers (µm) thick were prepared and stained with hematoxylin and eosin [20]. The slides were mounted with Entellan (Merck, Darmstadt, Germany). The obtained material was visualized in an Olympus BX-51 photomicroscope (Miami, FL, USA.), in the 20 and 40× objectives, coupled to the Olympus Computerized Image Analyzer System. The selected images were photographed for further morphological analysis using the Olympus Cellsens 1.14 software (Tokyo, Japan). Architecture, shape, nuclei position and analysis of possible modifications in the histological sections were observed, as well as the homogeneity of the fibers and presence of inflammatory infiltrate.

The sarcomere length was determined as described by [21]. From each raw sample, 0.5 g sub-samples were obtained and placed in a 50 mL falcon tube, to which 15 mL of 1.328% potassium iodide and 15 mL of 0.596% potassium chloride were added. Subsequently, the samples were homogenized in Ultra-turrax (Marconi MA102, Marconi Equipamentos Para Laboratórios Ltd., Piracicaba, São Paulo, Brazil) 15,000 rpm for 30 s. The slides were prepared with a drop of homogenate just before the reading. The readings were performed under a microscope (Novel BM2100, Nanjing Jlangnan Novel Optics., Ltd., China, People’s Republic) with phase contrast at 1000× magnification (100× objective, 10× eyepiece). The sarcomere length was expressed in µm.

### 2.5. Visual Affective Testing

**Figure 1:**
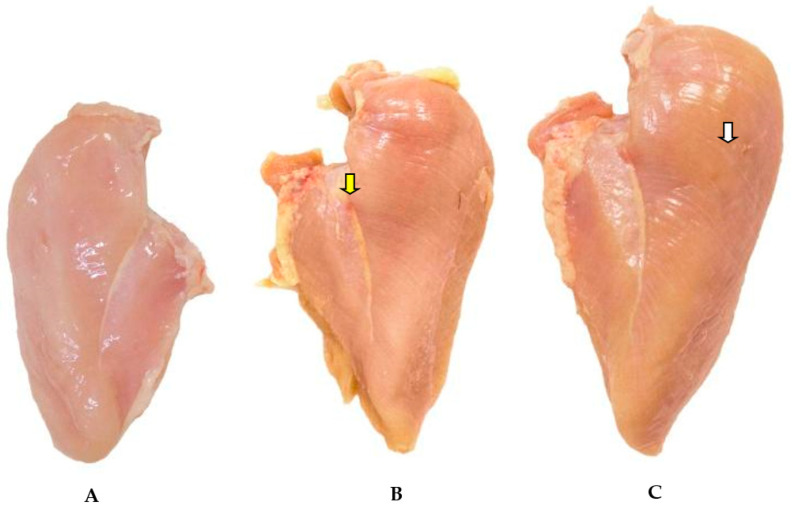
Normal *Pectoralis major* muscle (**A**), and *Pectoralis major* muscle affected by the moderate (**B**) and severe (**C**) degree of wooden breast myopathy (WBM) in Cobb broiler chicken. There are reddish (petechiae) and hardened areas in the middle and lower portions of the muscle (yellow arrow), as well as whitish striations in the upper quadrant of the muscle part (white arrow).

Visual affective testing was carried out to assess the differences perceived by the consumer on photos of fresh chicken breasts affected by WBM (Figure 1). A simple questionnaire containing personal data, chicken consumption and whether the consumer would buy the visualized product on the photo was applied. One hundred and twenty evaluators were recruited from supermarkets in the region of Jaboticabal/SP. The questionnaire is going to be published as a Supplementary Material.

### 2.6. Statistical Analysis

The data obtained in the physical-chemical analyses were analyzed using a completely randomized design (CRD) with 20 repetitions. Results were analyzed using the General Linear Models procedure of Statistical Analysis System (SAS Institute Inc. 2002–2003, Cary, NC, USA). All data were tested by analysis of variance (ANOVA) and compared by Tukey test at a significance level of 5%.

## 3. Results

### 3.1. Histological Analysis

The pectoral muscle of birds affected by moderate and severe degrees of WBM showed a general pale color with irregular, reddish hardened areas (presence of petechiae and suffusions), generally characterized as superficial lesions (Figure 1).

**Figure 2:**
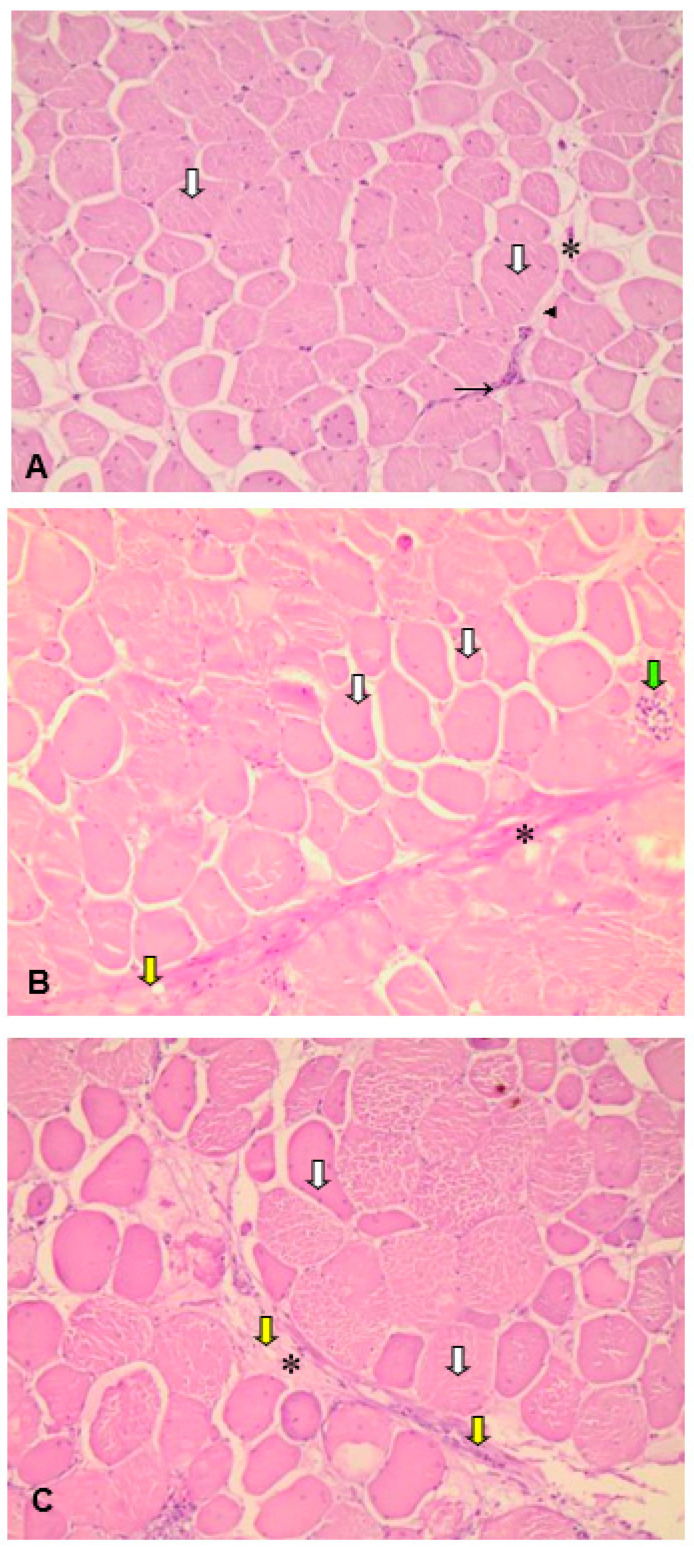
Photomicrographs of histological cross sections of *Pectoralis major* muscle from broilers affected by wooden breast (*n* = 20, for each group). (**A**) Normal. Muscle fiber (white arrow); perimysium (*); extracellular matrix (arrowhead); fibroblasts (black arrow). (**B**) Moderate aspect of the wooden breast. Muscle fiber (white arrow); perimysium (*); degenerated muscle fiber infiltrated by inflammatory cells (green arrow); adipocytes (yellow arrow). (**C**) Severe aspect of wooden breast. Muscle fiber (white arrow), perimysium (*), adipocytes (yellow arrows). HE, 200×.

In Figure 2A–C, skeletal muscle fibers of eccentric nuclei and dense non-modeled connective tissue, characterized as a perimysium that gathers skeletal muscle fibers in fascicles, were observed.

In the group of broiler chickens with healthy pectoral muscle, there was a relative homogeneity in the aspect of normal colored and polygonal muscle fibers. The perimysium was thinner compared to the moderate and severe groups, showing the extracellular matrix and fibroblasts, characteristic of interstitial or filling connective tissue (Figure 2A).

Both moderate and severe degrees of myopathy were characterized by the loss of the polygonal aspect of muscle fibers. The fibers of *Pectoralis major* muscle in broilers affected by the moderate degree of WBM showed heterogeneity in their size and increase in the thickness of interstitial connective tissue with a slight increase in the number of adipocytes. Degenerated fibers were also observed, infiltrated by inflammatory cells, mainly heterophiles and macrophages (Figure 2B).

The severe degree of WBM showed greater discrepancy in the size of muscle fibers and significant hypertrophy of these (Figure 2C).

### 3.2. Visual Affective Test

The visual affective test (*n* = 120) had a rejection rate greater than 82% for chicken breasts affected by WBM due to yellowing (8.67%), abnormality (1.76%), color (52.59%), fat (13.43%), appearance (12.97%), inflammation (2.67%), fibers (2.59%) and questionable appearance (2.59%).

### 3.3. Physical Analysis

Fillets affected by wooden breast myopathy showed greater weight (*p* < 0.001), greater length (*p* = 0.011) and width (*p* = 0.001), being that severe-degree samples were heavier among all evaluated groups (Table 1).

Chicken meat affected by the severe degree of WBM showed higher (*p* < 0.0001) pH (6.33) than chicken meat classified as normal (6.08) and moderate-degree (6.07) (Table 2).

The meat affected by the severe degree of WBM showed higher (*p* < 0.05) values of L* (65.19) and b* (3.59) on the external surface in relation to samples classified as normal (60.86 and 0.11, respectively) or as a moderate degree (60.23 and 1.59, respectively) (Table 2). Regarding the internal surface, the meat affected by the severe degree of WBM showed higher (*p* < 0.05) values of L* (62.05) and b* (5.16) than samples classified as moderate-degree (57.89 and 2.95, respectively) and normal samples (59.79 and 2.76, respectively). There was no significant difference (*p* > 0.05) between normal chicken samples and those affected by WBM as to the red intensity on external and internal surfaces.

Samples from broilers with a severe degree of WBM showed less water retention capacity and greater cooking loss than meat from normal chickens. As for meat’s tenderness, no significant differences (*p* > 0.05) were found between normal chicken samples and those affected by the myopathy (Table 3).

### 3.4. Chemical Analyses

The severe-degree samples of WBM showed a lower (*p* < 0.0001) myofibrillar fragmentation index (MFI) compared to normal samples and samples affected by the moderate degree of the condition (Table 4), a result that confirms the hardness of raw severe-degree samples.

Although a higher content of insoluble and total collagen was found in WBM, there were no significant differences (*p* > 0.05) in shear force in cooked chicken samples.

The samples with severe degree of WBM showed greater sarcomere length than samples with moderate degree and normal samples (*p* = 0.0005).

The samples with severe degree of WBM showed lower (*p* = 0.0002) fat concentration, greater (*p* < 0.0001) moisture and lower (*p* = 0.037) cholesterol concentration than normal samples and samples with moderate degree WBM (Table 5). There was no significant difference (*p* > 0.05) for the samples’ concentrations of protein and mineral matter and lipid oxidation.

There was an effect (*p* < 0.05) in the total concentration of saturated fatty acids (SFA) and in the concentrations of heptadecanoic (C17:0), stearic (C18:0), myristoleic (C14:1), γ linolenic (C18:3n6) and arachidonic (C20:4n6) acids (Table 6).

Broiler meat samples presented similar amounts of SFA and PUFA, although only the former showed significant difference: the severe degree of WBM showed a lower (*p* = 0.036) level of γ-linolenic acid.

There were no differences (*p* > 0.05) between normal chicken samples and those affected by WBM regarding the amount of EPA + DHA and the ω6/ω3 ratio.

## 4. Discussion

The present study aimed to characterize the effects of wooden breast myopathy (WBM) on quality of broiler chicken breast meat.

The heaviest weight found in chicken fillets affected by WBM was consistent with other studies [22,23,24] and may be associated with selection of animals for rapid growth and deposition of muscle mass, which exceeds the animals’ muscle support capacity [25,26]. Similar results were found by the authors of [27], who evaluated the impact of emerging myopathies on meat quality and concluded that normal breasts were the lightest and WBM meat was the heaviest, associating in addition to the factors already mentioned, breast weight and thickness, and older slaughtering age of the birds. Bowker et al. [28] reported that the fillet weight seemed to have less impact on average white striping score compared to WBM score after finding greater correlation coefficients between weight and WBM myopathy compared to white striping myopathy.

Chicken meat affected by the severe degree of WBM showed higher pH than chicken meat classified as normal and moderate-degree as also described in [29,30]. According to the authors of [31] the highest pH values observed in samples affected by WBM could be associated with glycogen storage, breast muscle weight and the correlation between these two variables, causing the larger breast to present reduced glycolytic potential, which results in a higher final pH. Other authors [32,33] pointed out that the less reduced final pH is also probably due to the energy status and changes in muscle metabolic pathways, where the amount of lactic acid produced during rigor mortis was not enough to reduce the pH of chicken fillets affected by WBM, especially in the severe-degree samples.

The color evaluation’s results indicate that samples affected by the myopathy (mainly in the severe-degree samples) are pale and yellowish in comparison to normal samples. This may be explained by changes in the muscular tissue after “tissue degeneration”, which causes greater moisture in the muscles affected by the low water retention capacity and severe fibrosis found in the fillets [25,30,34].

The muscles affected by WBM may exhibit reduced water retention capacity due to myodegeneration [35]. As we have observed, the meat affected by this condition has technological properties that are inferior to those of normal meat, such as reduced water retention capacity and texture changes [35]. These properties modify not only the processing, but the final quality of the meat as a whole [25], which may be associated with the characteristic hardness of cuts with this myopathy. However, that was not observed in this study, although WBM may have caused a higher value of L* in chicken fillets affected by the severe degree (Table 2). Cooking losses, which result in less juicy and less tender meat [36], were greater in samples with WBM compared to samples from normal chickens. Histological evaluation was performed in our study (Figure 2) and, similar to the literature [2,37,38], revealed structural changes in muscular tissue from chicken breasts affected by WBM, such as muscle fiber degeneration that contributes to the reduction of water retention capacity, causing greater cooking losses associated with the accumulation of intramuscular fat, which may have caused a higher b* value on the outer and inner surface of chicken fillets in samples affected by WBM, especially in the severe-degree samples (Table 2).

The myofibrillar fragmentation index is directly related to softness, with higher MFI values being correlated with lower shear force and greater softness [18,39]. There are no established standards for myofibrillar fragmentation index in chicken meat, which makes it more difficult to relate it to tenderness [40].

Regarding the collagen concentration, the results indicated that breast samples affected by the myopathy had higher concentrations of insoluble and total collagen when compared to normal samples, which may contribute to tissue stiffness, softness reduction and meat quality impairment [41]. Fibrosis probably led to an increase in collagen concentration [41]. The increase in the stiffness of the muscle affected by WBM is associated not only to an increase in the collagen concentration, but also to structural and extension characteristics of the fibrous connective tissue, fibril diameter, reticulation, fibril density and other structural characteristics [42].

Although a higher content of insoluble and total collagen was found in WBM, there were no significant differences (*p* > 0.05) in shear force in cooked chicken samples, possibly due to the denaturation and solubilization of cross-linked collagen that occur at temperatures between 53 °C and 63 °C [43].

The result of sarcomere length suggests that the hardness observed in fillets affected by WBM in other studies occurs due to other circumstances besides the shortening of sarcomeres [41]. Tijare et al. [44] reported that sarcomeres affected by WBM tend to be longer than normal breast fillets. Some studies suggest that there is a direct relationship between sarcomere length and meat tenderness, being that the greater the sarcomere length the greater the meat tenderness [45,46]. This relationship should be used in chicken fillets affected by WBM.

The lower fat concentration observed in chicken samples affected by the moderate and severe degrees of WBM may have provided the difference observed between samples for lipid oxidation (*p* > 0.05). Increase in the fat concentration also increases the cholesterol present in the meat [47]. However, in this study, we expected that, when decreasing the fat concentration, the total cholesterol concentration would also decrease, but this reduction was not observed in samples affected by the moderate degree of WBM. Despite these results, the cholesterol level of all samples is within the normal range, ranging from 58 to 104 mg/100 g, depending on the type of chicken meat used [48].

The γ-linolenic acid is a PUFA of the ω6 family, essential for maintaining the normal function of cell membranes, in addition to being important for the maintenance of brain functions and the transmission of nerve impulses [41]. Some studies report that this fatty acid is used in the biosynthesis of arachidonic acid, a relationship that was not observed in our research, considering that, among the samples, we obtained a lower concentration of γ-linolenic acid and a higher concentration of arachidonic acid in samples affected by the severe degree of WBM.

It is assumed that EPA and DHA fatty acids are important because they can attenuate the effects of the inflammatory process by decreasing the synthesis of eicosanoids [41]. In this study, the ω6/ω3 ratio was approximately 12:1, which exceeded the 6:1 recommendation [49], since PUFAs are important for cardiovascular health.

Similar to the results in [2], we observed that the muscular surface was often covered by a thin layer of clear or slightly cloudy, moderately viscous material in addition to dispersed petechiae and suffusions, with accumulation of small hemorrhages in some points. Zhang et al. [5] evaluated light microscopic structures of meat batter gels from chicken broiler breasts with different wooden breast conditions and examining the histological sections exhibited many differences linked to the alteration in muscle, and suggested that the occurrence of wooden breast abnormality led to increased moisture, fat, and collagen contents coupled with reduced total protein and ash levels.

In the moderate and severe groups, the presence of white striations parallel to the muscle fibers was observed. The severity was greater the more severe the wooden breast myopathy was. The increase in striations was also associated with greater injury severity in the pectoral muscles of broilers by [50].

According to [2], degenerated muscle fibers were surrounded by loosely organized connective tissue and rich in collagen. Miodegeneration, accompanied by regeneration, is significantly associated with the wooden breast [51]. The expression of specific regulatory factors of muscle protein transcription for proliferation and differentiation of cells that promote muscle regeneration in response to muscle damage depends on the type of fast-growing commercial strain [41]. This suggests that the etiology of wooden breast myopathy may vary between broiler strains [38].

According to [52], when the increase in the size of muscle fibers is not accompanied by adequate nutritional support, it can result in intermediate metabolic stress, due to the difficulty in diffusing oxygen in the muscle tissue. The inflammatory infiltrate is also observed next to the perimysium (Figure 2C). On the other hand, muscle fibers with an abnormal polygonal profile (rounded fibers) were found in correspondence to diffuse hardened areas in WBM breasts in a similar study by [53], where an increase in intramuscular fat was observed.

Histological results of the present work are similar to those presented by [54] who raised the hypothesis that the damage to mitochondria mediated by hypoxia serves as the first step in Wooden Breast pathogenesis, followed by muscle pathology such as hypertrophy and fibrosis. Praud et al. [55] worked with molecular phenotyping of white striping and wooden breast myopathies in chicken and propose the use of histological and molecular tools allowing for precise quantification of the different lesions present in muscles affected by white striping or wooden breast. The same authors say that molecular phenotyping will allow progress in understanding the etiology of these defects but also by refining the diagnosis of injuries accelerate the development of non-invasive prediction tools at the service of breeders and producers. The present work is an additional contribution in line with recent reports [54,55].

As a consequence of the results of visual affective test, chicken breast affected by wooden breast myopathy does not present sensory patterns of direct commercialization, being better used for the production of possible by-products.

## 5. Conclusions

Samples affected by wooden breast myopathy are larger and heavier. Chicken samples affected by the severe degree of WBM present inferior physical quality compared to normal chicken meat, which can be harmful to the processing of poultry meat. In addition, the severe degree of WBM has normal levels for fatty acids, besides having a higher level of arachidonic acid, an important ω6 fatty acid that reduces cholesterol levels. Chicken breasts with the myopathy have a lower concentration of fat than normal breasts. Myodegeneration caused by WBM has muscle fiber atrophy as a compensatory mechanism in moderate and severe degrees in broilers. The loss of the polygonal aspect, the heterogeneity of most muscle fibers, the increase in interstitial connective tissue and the inflammatory infiltrate characterize the histological aspects of the breast meat affected by moderate and severe degrees of WBM. Additionally, in the visual affective test of fresh chicken breast, the consumers disapproved of the breasts affected by WBM, regardless of the degree of involvement.

## Figures and Tables

**Table 1:** Weight, length and breadth of breasts from broilers affected by wooden breast myopathy.

Variables	Normal (*n* = 20)	Moderate (*n* = 20)	Severe (*n* = 20)	*p*-Value
Weight (g)	523.13 ± 18.58 ^c^	600.88 ± 18.58 ^b^	695.82 ± 18.58 ^a^	<0.001
Length (cm)	18.34 ± 0.25 ^b^	19.36 ± 0.25 ^a^	19.17 ± 0.25 ^a^	0.011
Width (cm)	10.90 ± 0.15 ^b^	11.43 ± 0.15 ^a^	11.72 ± 0.15 ^a^	0.001

^a–c^ Means followed by distinct letters in the lines differ from each other by the Tukey test (*p* < 0.05).

**Table 2:** Mean values and standard deviation of luminosity (L*), intensity of red (a*) and yellow (b*) of the breast meat of broilers affected by WBM.

Variables	Normal (*n* = 20)	Moderate (*n* = 20)	Severe (*n* = 20)	*p*-Value
pH	6.08 ± 0.04 ^b^	6.07 ± 0.03 ^b^	6.33 ± 0.54 ^a^	<0.001
Outer surface				
L*	60.86 ± 0.97 ^b^	60.23 ± 0.68 ^b^	65.19 ± 1.08 ^a^	0.002
a*	1.02 ± 0.42	1.18 ± 0.22	0.92 ± 0.20	0.686
b*	0.11 ± 0.36 ^c^	1.59 ± 0.28 ^b^	3.59 ± 0.52 ^a^	<0.001
Inner Surface				
L*	59.79 ± 0.85 ^ab^	57.89 ± 1.03 ^b^	62.05 ± 1.07 ^a^	0.033
a*	1.01 ± 0.29	0.94 ± 0.20	1.41 ± 0.50	0.677
b*	2.76 ± 0.15 ^b^	2.95 ± 0.34 ^b^	5.16 ± 0.53 ^a^	0.002

^a–c^ Means followed by distinct letters in the lines differ from each other by the Tukey test (*p* < 0.05).

**Table 3:** Mean values and standard deviation of water retention capacity (WRC), cooking weight loss (CWL) and shear force by Warner–Bratzler (WB) and Meullenet–Owens Razor Shear (MORS) methods of breast meat from broilers affected by WBM.

Variables	Normal (*n* = 20)	Moderate (*n* = 20)	Severe (*n* = 20)	*p*-Value
WRC (%)	71.89 ± 0.89 ^a^	72.51 ± 0.75 ^a^	68.92 ± 1.02 ^b^	0.027
CWL (%)	22.54 ± 0.67 ^b^	26.25 ± 0.59 ^a^	26.27 ± 0.77 ^a^	<0.001
WB (N)	29.70 ± 4.49	37.50 ± 3.31	28.81 ± 5.38	0.149
MORS (N)	11.58 ± 1.56	9.82 ± 0.48	9.83 ± 0.47	0.554

^a,b^ Means followed by distinct letters in the lines differ from each other by the Tukey test (*p* < 0.05).

**Table 4:** Mean values and standard deviation of the myofibrillar fragmentation index (MFI), collagen and sarcomere length of breast meat from broilers affected by WBM.

Variables	Normal (*n* = 20)	Moderate (*n* = 20)	Severe (*n* = 20)	*p*-Value
MFI	80.45 ± 6.22 ^a^	92.99 ± 3.79 ^a^	64.51 ± 3.81 ^b^	<0.0001
Soluble collagen (%)	0.13 ± 0.02	0.17 ± 0.02	0.18 ± 0.02	0.1490
Insoluble collagen (%)	0.16 ± 0.02 ^b^	0.26 ± 0.02 ^a^	0.30 ± 0.04 ^a^	0.0006
Total collagen (%)	0.29 ± 0.03 ^b^	0.43 ± 0.04 ^a^	0.48 ± 0.05 ^a^	0.0040
Sarcomere (µm)	1.50 ± 0.02 ^b^	1.49 ± 0.02 ^b^	1.66 ± 0.04 ^a^	0.0005

^a,b^ Means followed by distinct letters in the lines differ from each other by the Tukey test (*p* < 0.05).

**Table 5:** Mean values and standard deviation of crude protein, fat, moisture and mineral matter concentrations; lipid oxidation (TBARS) and cholesterol of breast meat from broilers affected by WBM and stored for 30 days at −20 °C.

Variables	Normal (*n* = 20)	Moderate (*n* = 20)	Severe (*n* = 20)	*p*-Value
Protein (%)	24.64 ± 0.65	23.31 ± 0.45	22.97 ± 0.48	0.1260
Fat (%)	3.79 ± 0.33 ^a^	2.76 ± 0.22 ^b^	2.17 ± 0.13 ^c^	0.0002
Moisture (%)	73.66 ± 0.24 ^b^	74.20 ± 0.15 ^b^	76.41 ± 0.20 ^a^	<0.0001
Mineral matter (%)	1.77 ± 0.17	1.74 ± 0.16	1.46 ± 0.15	0.3230
TBARS (mg MDA/kg)	0.613 ± 0.048	0.588 ± 0.029	0.647 ± 0.064	0.6830
Total cholesterol (mg/100 g)	84.93 ± 1.01 ^ab^	86.56 ± 1.04 ^a^	82.49 ± 1.09 ^b^	0.0370

^a–c^ Means followed by distinct letters in the lines differ from each other by the Tukey test (*p* < 0.05).

**Table 6:** Fatty acid composition (% of total fatty acids) of breast meat fat from Cobb MX broilers affected by WBM.

Fatty Acids	Treatments (*n* = 20)			*p*-Value
	Normal	Moderate	Severe	
SFA	32.10 ± 0.10 ^b^	32.45 ± 0.34 ^b^	33.31 ± 0.18 ^a^	0.0001
MUFA	35.91 ± 1.04	35.71 ± 0.89	34.00 ± 0.33	0.087
PUFA	32.00 ± 0.98	31.85 ± 0.73	32.70 ± 0.30	0.490
C12:0	0.03 ± 0.01	0.03 ± 0.01	0.03 ± 0.01	0.970
C14:0	0.46 ± 0.02	0.50 ± 0.01	0.47 ± 0.01	0.136
C15:0	0.077 ± 0.002	0.073 ± 0.002	0.077 ± 0.003	0.499
C16:0	24.02 ± 0.23	24.57 ± 0.22	24.44 ± 0.16	0.227
C17:0	0.117 ± 0.006 ^ab^	0.105 ± 0.003 ^b^	0.123 ± 0.004 ^a^	0.013
C18:0	7.32 ± 0.25 ^b^	7.09 ± 0.19 ^b^	8.08 ± 0.10 ^a^	0.0007
C20:0	0.073 ± 0.002	0.073 ± 0.003	0.077 ± 0.002	0.499
C14:1	0.093 ± 0.010 ^ab^	0.103 ± 0.005 ^a^	0.087 ± 0.003 ^b^	0.043
C16:1	3.55 ± 0.33	3.77 ± 0.18	3.25 ± 0.10	0.061
C17:1	0.05 ± 0.01	0.05 ± 0.01	0.05 ± 0.01	0.615
C18:1n9c	30.01 ± 0.73	29.57 ± 0.70	28.37 ± 0.30	0.083
C18:1n7	1.94 ± 0.07	1.97 ± 0.08	1.99 ± 0.03	0.855
C20:1n9	0.26 ± 0.01	0.25 ± 0.01	0.26 ± 0.01	0.958
C18:2n6	26.11 ± 0.70	26.19 ± 0.58	26.07 ± 0.11	0.976
C18:2c9, t11	0.062 ± 0.003	0.058 ± 0.001	0.050 ± 0.009	0.08
C20:2	0.45 ± 0.05	0.42 ± 0.05	0.48 ± 0.03	0.501
C18:3n6	0.180 ± 0.003 ^a^	0.192 ± 0.014 ^ab^	0.163 ± 0.006 ^b^	0.036
C18:3n3	1.66 ± 0.06	1.71 ± 0.05	1.62 ± 0.03	0.363
C20:3n6	0.38 ± 0.05	0.38 ± 0.04	0.45 ± 0.03	0.380
C20:3n3	0.052 ± 0.006	0.047 ± 0.008	0.048 ± 0.003	0.850
C20:4n6	1.85 ± 0.27 ^ab^	1.68 ± 0.20 ^b^	2.34 ± 0.15 ^a^	0.041
C22: 4n6 (DTA)	0.58 ± 0.09	0.54 ± 0.05	0.72 ± 0.06	0.067
C20:5n3 (EPA)	0.11 ± 0.01	0.12 ± 0.02	0.13 ± 0.01	0.461
C22:5n3 (DPA)	0.38 ± 0.05	0.35 ± 0.04	0.46 ± 0.03	0.118
C22:6n3 (DHA)	0.19 ± 0.03	0.17 ± 0.02	0.18 ± 0.01	0.844
EPA + DHA	0.30 ± 0.04	0.28 ± 0.04	0.31 ± 0.01	0.819
ω6	29.10 ± 0.86	28.98 ± 0.65	29.74 ± 0.27	0.488
ω3	2.39 ± 0.09	2.39 ± 0.06	2.43 ± 0.02	0.709
ω6/ω3	12.19 ± 0.15	12.13 ± 0.15	12.22 ± 0.07	0.867

^a–b^ Means followed by different lowercase letters on the same line differ from each other by the Tukey test (*p* < 0.05). SFA, saturated fatty acids; MUFA, monounsaturated fatty acids; PUFA, polyunsaturated fatty acids; DTA, docosatetraenoic acid; EPA, eicosapentaenoic acid; DPA, docosapentaenoic acid; DHA, docosahexaenoic acid; ω6/ω3—ω6/ω3 ratio.

## Data Availability

This data can be found here: https://repositorio.unesp.br/bitstream/handle/11449/191219/oliveira_rf_dr_jabo.pdf?sequence=5&isAllowed=y.

## Supplementary Materials

The following are available online at https://www.mdpi.com/2076-2615/11/3/596/s1, Figure S1: Visual affective test sheet.

## Abbreviations

WBM	Wooden Breast Myopathy
UNESP	Paulista State University
cm	Centimeter
L*	Luminosity
a*	Intensity of red
b*	Intensity of yellow
pH	Hydrogen potential
WHC	Water Holding Capacity
CWL	Cooking Weight Loss
MORS	Meullenet-Owens Razor Shear
TBARs	Thiobarbituric Acid reactive Substances
KOH	Potassium hydroxide
MFI	Myofibrillary Fragmentation Index
µm	micrometers
CRD	Completely Randomized Design
SAS	Statistical Analysis System
ANOVA	Analysis of Variance
WB	Warner–Bratzler
SFA	Saturated Fatty Acids
MUFA	Monounsaturated Fatty Acids
PUFA	Polyunsaturated Fatty Acids
DTA	Docosatetraenoic Acid
EPA	Eicosapentaenoic Acid
DPA	Docosapentaenoic Acid
DHA	Docosahexaenoic Acid
FAPESP	Foundation for Research Support of the State of São Paulo
CAPES	Coordenação de Aperfeiçoamento de Pessoal de Nível Superior-Brasil
