# Supplementary material for: Physical, Chemical and Histological Characterization of Pectoralis major Muscle of Broilers Affected by Wooden Breast Myopathy
Source: Animals (Basel). 2021 Feb 24;11(3):596. doi: 10.3390/ani11030596 (PMC7996277; doi:10.3390/ani11030596)
Supplement: Supplementary file 1 [file animals-11-00596-s001.pdf]

SENSORIAL ANALYSIS - CHICKEN WOODEN BREAST

Gender ( ) M ( ) F

Age group

( ) Under 21 years old
( ) 21 to 30 years old
( ) 31 to 40 years old
( ) 41 to 50 years old
( ) 51 to 60 years old
( ) 61 to 70 years old
( ) Above 70 years old

Education level

( ) Higher
( ) Higher (incomplete)
( ) 2nd grade
( ) 1º grade
( ) Non-literate student

Preferred product type

( ) Packed
( ) In bulk / by weight

Most frequently purchased product

( ) Whole
( ) Breast
( ) Breast fillet
( ) Thigh
( ) Leg quarter with back
( ) Wings
( ) Other parts

When you go shopping, you decide for:

( ) Price
( ) Brand
( ) Appearance
( ) Practicality
( ) Pack size
( ) Nutricional Value

Purchase frequency

( ) Every day
( ) Every week
( ) Once a month
( ) Every three months

Frequency of consumption

( ) Every day
( ) Once a week
( ) 3 times a week
( ) Every 15 days
( ) Once a month
( ) Less than once a month

How many people will consume this product? _____________

PHOTO 1

Would you buy this chicken breast? Yes ( ) No ( )
If no, why?

PHOTO 2

Would you buy this chicken breast? Yes ( ) No ( )
If no, why?

PHOTO 3

Would you buy this chicken breast? Yes ( ) No ( )
If no, why?

Any comments on the appearance of the photo samples?
____

Figure. Visual affective test sheet
